# "Three-in-One" Multi-Scale Structural Design of Carbon Fiber-Based Composites for Personal Electromagnetic Protection and Thermal Management

**DOI:** 10.1007/s40820-023-01144-z

**Published:** 2023-07-10

**Authors:** Ming Zhou, Shujuan Tan, Jingwen Wang, Yue Wu, Leilei Liang, Guangbin Ji

**Affiliations:** 1 College of Materials Science and Technology, Nanjing University of Aeronautics and Astronautics, No. 29 Yudao Street, Nanjing, 210016 People’s Republic of China; 2 School of Electronic Science and Engineering, Nanjing University, Nanjing, 210093 People’s Republic of China

**Keywords:** Electromagnetic shielding, Multi-scale design, One-dimensional materials, Carbon fiber, Thermal management

## Abstract

Wearable devices with efficient thermal management and electromagnetic interference (EMI) shielding are highly desirable for improving human comfort and safety. Herein, a multifunctional wearable carbon fibers (CF) @ polyaniline (PANI) / silver nanowires (Ag NWs) composites with a “branch-trunk” interlocked micro/nanostructure were achieved through "three-in-one" multi-scale design. The reasonable assembly of the three kinds of one-dimensional (1D) materials can fully exert their excellent properties *i.e.*, the superior flexibility of CF, the robustness of PANI, and the splendid conductivity of AgNWs. Consequently, the constructed flexible composite demonstrates enhanced mechanical properties with a tensile stress of 1.2 MPa, which was almost 6 times that of the original material. This is mainly attributed to the fact that the PNAI (branch) was firmly attached to the CF (trunk) through polydopamine (PDA), forming a robust interlocked structure. Meanwhile, the composite possesses excellent thermal insulation and heat preservation capacity owing to the synergistically low thermal conductivity and emissivity. More importantly, the conductive path of the composite established by the three 1D materials greatly improved its EMI shielding property and Joule heating performance at low applied voltage. This work paves the way for rational utilization of the intrinsic properties of 1D materials, as well as provides a promising strategy for designing wearable electromagnetic protection and thermal energy management devices.

**Supplementary Information:**

The online version contains supplementary material available at 10.1007/s40820-023-01144-z.

## Highlights

- A multi-scale structural carbon fiber-based composite was synthesized through the assembly of one-dimensional materials.
- The construction of multiple conductive networks makes the composite have a strong EMI shielding performance of 73.9 dB.
- The reasonable design endows the composite with excellent positive and passive thermal management properties.

**Supplementary Information:**

The online version contains supplementary material available at 10.1007/s40820-023-01144-z.

## Graphical abstract

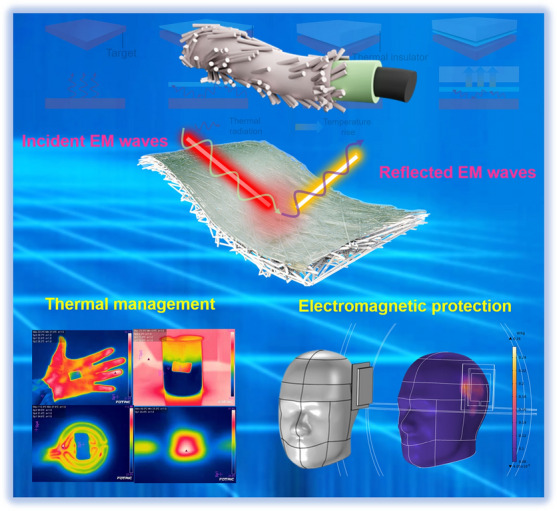

## Introduction

The blossom of information technology has led mankind into the intelligent age, which has put forward higher requirements for wearable devices, *i.e.*, functional integration and system stability. Following the trend of the progress of the times, various innovative wearable electronics integrated with functions, such as health management [1], motion monitoring [2, 3], thermal energy control [4], electromagnetic interference (EMI) shielding [5, 6] and so on, have been invented to meet the demands of human beings for a better life. Notably, an important function of wearable devices is to maintain a relatively stable and comfortable body temperature to keep the body functioning, especially in areas with cold weather and insufficient sunshine [7]. Nevertheless, traditional thermal insulation strategies, such as wearing heavy clothing, although effective, could hinder human movement and reduce comfort. In addition, the integration of wearable electronics will worsen the electromagnetic (EM) environment around users, which interferes with normal electronic equipment and has a negative impact on human health through the "thermal effect" and "non-thermal effect" of EM waves [8–13]. Consequently, functionalized flexible wearable composites, which can manage the thermal energy between people and environment, and provide effective EM protection, have attracted extensive attention. However, there are many challenges in the functionalization process of wearable devices. For example, current material designs for multifunctional integration tend to be complex, and a single material cannot take on multiple roles, thus requiring rational component and structural design.

Multi-level structure design on a nano-micro scale of materials is an effective strategy to achieve multifunctional integration, thus it is one of the focuses of current research [14–16]. Liu et al. [17] designed a multifunctional collagen fiber-based flexible wearable sensing composite with a nano-micro structure by combining *in-situ* polymerization, spraying technology, and impregnation method. The collagen fiber with a unique hierarchical structure provided supporting sites for conductive polymers and functional nanomaterials. Thus, the obtained composite showed superior flame-retardant performance, EMI shielding property, and photothermal conversion feature. Zhang and his co-works [18] prepared a flame-retardant hybrid foam using 0D ferroferric oxide nanoparticles, and 1D Ag NWs. The combination of magnetoelectric materials endowed the composite with excellent absorption-dominant EMI-shielding performance. In addition, the multilayer porous micro/nanostructure made the composite have great thermal insulation performance, which could be applied in the field of infrared stealth. As mentioned above, it is believed that the multi-level micro-nano structure design of the synthesized macro-composites not only inherits the physical and chemical characteristics of the individual units, but also realizes multiple properties or special functions by the unique structure.

1D materials such as carbon materials (carbon fiber, carbon nanotube), metal materials (copper nanowire, AgNWs), and polymers (polypyrrole, polyaniline (PANI)) have a wide application prospect in the fields of catalysis [19], energy [20] and EMI shielding [21] due to their merits of large aspect ratio, ideal electric and thermal characteristic [22]. For example, a laminated structural engineering strategy was proposed to synthesize the aerogel film by using carbon nanotubes [23]. Due to the layered porous structure with preferential orientation and continuous conductive path constructed by carbon nanotubes, the aerogel film exhibited ultra-high EMI shielding effectiveness with SSE (specific shielding effectiveness)/t of 200,647.9 dB cm^2^ g^−1^, which was superior to the aerogel-based materials reported at present. Celle et al. [24] developed a conductive and mechanically resistant AgNWs/carboxymethylcellulose (CMC) aerogel. The addition of CMC could effectively overcome the inferior mechanical performance of AgNWs, and the compression performance could be improved from 10.4 ± 0.9 kPa for Ag NWs aerogels to 740 ± 40 kPa for Ag NWs/CMC aerogel. It can be seen that it is feasible to obtain ideal properties by reasonable assembly of 1D materials.

**Fig. 1:**
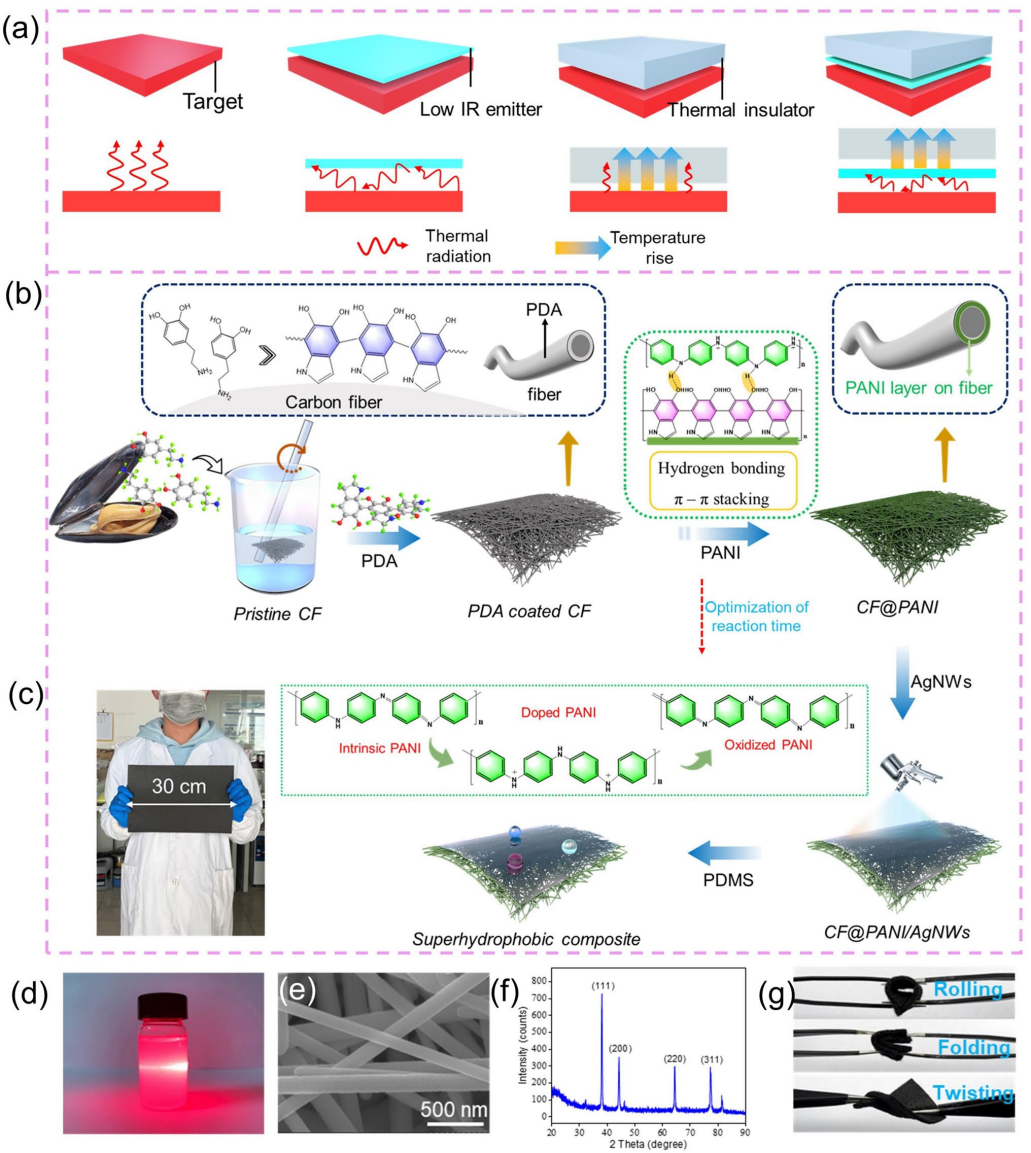
**a** Schematic diagram of the heat change mechanism of different materials. **b** Fabrication process of the CF@PANI/AgNWs composite. **c** Digital image of the prepared large-scale CF@PANI/AgNWs composite. **d** Typical Tyndall effect of the ethanol dispersion of AgNWs. **e** SEM image of the AgNWs. **f** XRD pattern of the prepared AgNWs. **g** Photograph of the CF@PANI/AgNWs composite with great flexibility, including rollability foldability, and twistability

In this work, a rational multi-level assembly design of 1D materials was proposed to integrate the carbon fiber, PANI nanofiber, and AgNWs. The unique “branch-trunk” interlocked micro/nanostructure was formed by the strong adhesive ability of polydopamine (PDA), greatly improving the mechanical performance of carbon fiber. More importantly, through the dual manipulation strategy of synergistically controlling thermal conductivity and infrared radiation (Fig. 1a), the heat management of the obtained composite was optimized, which could be applied in the field of thermal insulation and infrared stealth. Notably, the multi-level structure design could construct the conductive networks to boost the EMI shielding performance, and give the composite active thermal management performance by Joule heating. This work provides a feasible strategy for assembling 1D materials to obtain stably interlocked micro/nanostructure, demonstrating their great potential for EMI shielding and thermal control, which is highly promising for dual-function wearable devices with thermal energy management and electromagnetic protection.

## Experimental Section

### Materials

Carbon felt (CF) with a thickness of ~ 0.5 mm was bought from an online store. The density of the CF was about 0.149 g cm^−3^ and the diameter of carbon fibers constituting the CF was ~ 8 μm. Tris (hydroxymethyl)methyl aminomethane (C_4_H_11_NO_3_), dopamine hydrochloride (C_8_H_11_NO_2_ClH) and ammonium sulfate ((NH_4_)_2_S_2_O_8_, APS) were all purchased from Nanjing chemical reagent Co., Ltd. Aniline (C_6_H_5_NH_2_, AN) and isopropyl alcohol (C_3_H_8_O, IPA) were obtained from Shanghai Macklin Biochemical Co., Ltd. Silver nitrate (AgNO_3_) came from Sinopharm Chemical Reagent Co., Ltd. Polydimethylsiloxane (PDMS, DC184) was acquired from DOWSIL. All chemical reagents were analytically pure and used without further purification.

### Modification of CF Surface with PDA

The original CF was first ultrasonically washed in water and ethanol for 30 min to eliminate impurities and oil pollution attached to the surface and dried in the vacuum oven (60 ℃) for 1 h. Then the cleaned CF was immersed into the 200 mL Tris (hydroxymethyl)aminomethane solution with a concentration of 1.2 g L^−1^ and stirred for about 30 min. After that, a certain mass of dopamine hydrochloride (a concentration of 2 g L^−1^) was added to the solution and stirred away from light for 24 h. At the end of the reaction, the CF was washed several times with deionized water and dried in an oven at 60 ℃.

### Preparation of Superhydrophobic CF@PANI/AgNWs Composite

The modified CF was dipped into a 10 mL hydrochloric acid (HCl, 1 mol L^−1^) solution containing 1 mL AN for about 30 min. Then the HCl solution (1 mol L^−1^) containing APS with the same molar mass as aniline was added to the above solution. It is noted that the whole reaction process was carried out at about ~ 0 ℃. After a period of reaction, the samples were taken out and washed several times with deionized water. The CF@PANI composites were obtained after drying in an oven, which was denoted as PA0-PA4 according to the different polymerization time (0–4 h) of PANI. The spraying method was used to form a uniform AgNWs layer on the surface of the CF@PANI composite. And the synthesis of AgNWs was based on the reported work [25]. It is noted that the area density of AgNWs on the composite was about 663.7 mg m^−2^ according to the distance of the spray gun and the spraying area. For convenience, the sample sprayed with AgNWs was labeled as PAgx, where x was defined as the polymerization time of PANI.

The PDMS was selected as the hydrophobic agent to endow the composite material with waterproof performance. In a typical hydrophobic treatment process, the PDMS and its solidifying agent (the mass ratio is 10:1) were dissolved in 30 mL IPA, then the prepared CF@PANI/AgNWs composite was immersed into it for 1 h and cured at 80 ℃ for 12 h. Finally, the superhydrophobic CF@PANI/AgNWs composite was acquired.

### Characterizations

The internal structure and morphology of samples were observed by the FESEM (field-emission scanning electron microscopy, Hitachi S4800). The X-ray diffraction curves were acquired by an XRD (X-ray diffractometer, Bruker D8 ADVANCE) using Cu Kα radiation (*λ* = 1.5604 Å). An XPS (X-ray photoelectron spectrometer, PHI 5000 VersaProbe) was used to investigate the element state of samples. An FT-IR spectrometric analyzer (Nicolet 5700) with KBr pellets was employed to obtain the Fourier transform infrared spectrum. The electric conductivity of the samples was characterized by a four-point probe instrument (RTS-8). The infrared emissivity of the samples was recorded by an IR-2 dual-band infrared emissivity meter (Shanghai Institute of Technical Physics, Chinese Academy of Sciences). The water contact angle (WCA) was studied by the Contact angle tester (JC2000D7M). To evaluate the electromagnetic shielding property of samples in the frequency range of 8.2–12.4 GHz (X-band), a vector network analyzer (VNA, Agilent PNA N5244A) was used. According to the requirement of the waveguide method, the tested samples were cut to a size of 22.86 mm × 10.16 mm. The measured scattering parameters (*S*_11_, *S*_21_) were used to calculate the total SE (SE_T_), reflection SE (SE_R_), and absorption SE (SE_A_) based on the following formulas [26, 27]:

1 $$R = \left| {S_{11} } \right|^{2}$$

2 $$T = \left| {S_{21} } \right|^{2}$$

3 $$R + A + T = 1$$

4 $$SE_{R} = - 10\log \left( {1 - R} \right) = - 10{\text{log}}\left( {1 - \left| {S_{11} } \right|^{2} } \right)$$

5 $$SE_{A} = - 10\log \left( {T/\left( {1 - R} \right)} \right) = - 10\log \left( {|S_{21} |^{2} /\left( {1 - \left| {S_{11} } \right|^{2} } \right)} \right)$$

6 $$SE_{T} = SE_{R} + SE_{A}$$

where *A*, *R*, and *T* are the absorption coefficient, reflectivity coefficient, and transmissivity coefficient, respectively. Notably, multiple internal reflections (SE_M_) can be ignored when the SE_A_ exceeds 10 dB or the thickness of the material is larger than that of the skin depth [28].

## Results and Discussion

### Design Strategy and Structural Characterizations

The preparation processes of the CF@PANI/AgNWs composite are schematically shown in Fig. 1b. To activate the surface of the pristine CF, the PDA was chosen as the active agent due to its mild reaction, environmental friendliness, and strong adhesion to the substrate [29]. In addition, the PDA could provide a large number of oxygen-containing functional groups, which is beneficial for further modification [30]. As shown in Fig. S1, the pristine CF presented hydrophobic characteristics (Fig. S1a, b). After surface activation, the CF became hydrophilic, which is due to the hydrophilic groups contained in PDA (Fig. S1c, d). Then the functional layer of PANI was synthesized on the modified CF through in-situ polymerization. It is noted that the electrical performance of the PANI can be tuned by the reaction time, which has a great impact on the EMI shielding property of the composite [31]. Therefore, the suitable polymerization time of PANI needs to be chosen in this work. After that, the AgNWs (with a diameter of ca.140 nm and a length of ca. 18 μm, Figs. 1e and S2) were sprayed on the surface of PANI. From Fig. 1d, it can be found that a bright path formed in the prepared AgNWs dispersion due to the scattering behavior of laser (a typical Tyndall effect), indicating the good dispersion of AgNWs in ethanol [32]. The XRD spectrum (Fig. 1f) presents some characteristic peaks located at 38°, 44.9°, 64.9°, and 77.9°, which attributes to the (111), (200), (220), and (311) planes of the face-centered-cubic (FCC) AgNWs [33]. All the analysis indicates that AgNWs had been successfully synthesized. Ultimately, the as-required composite was obtained by coating PDMS on its surface. Through the above facile method, we can easily obtain large-sized composite (Fig. 1c) or even industrial-grade products. Besides, the prepared composite could maintain its integrity even under the condition of rolling, folding, and twisting (Fig. 1g), demonstrating its great flexibility, which may be greatly suitable for wearable devices.

**Fig. 2:**
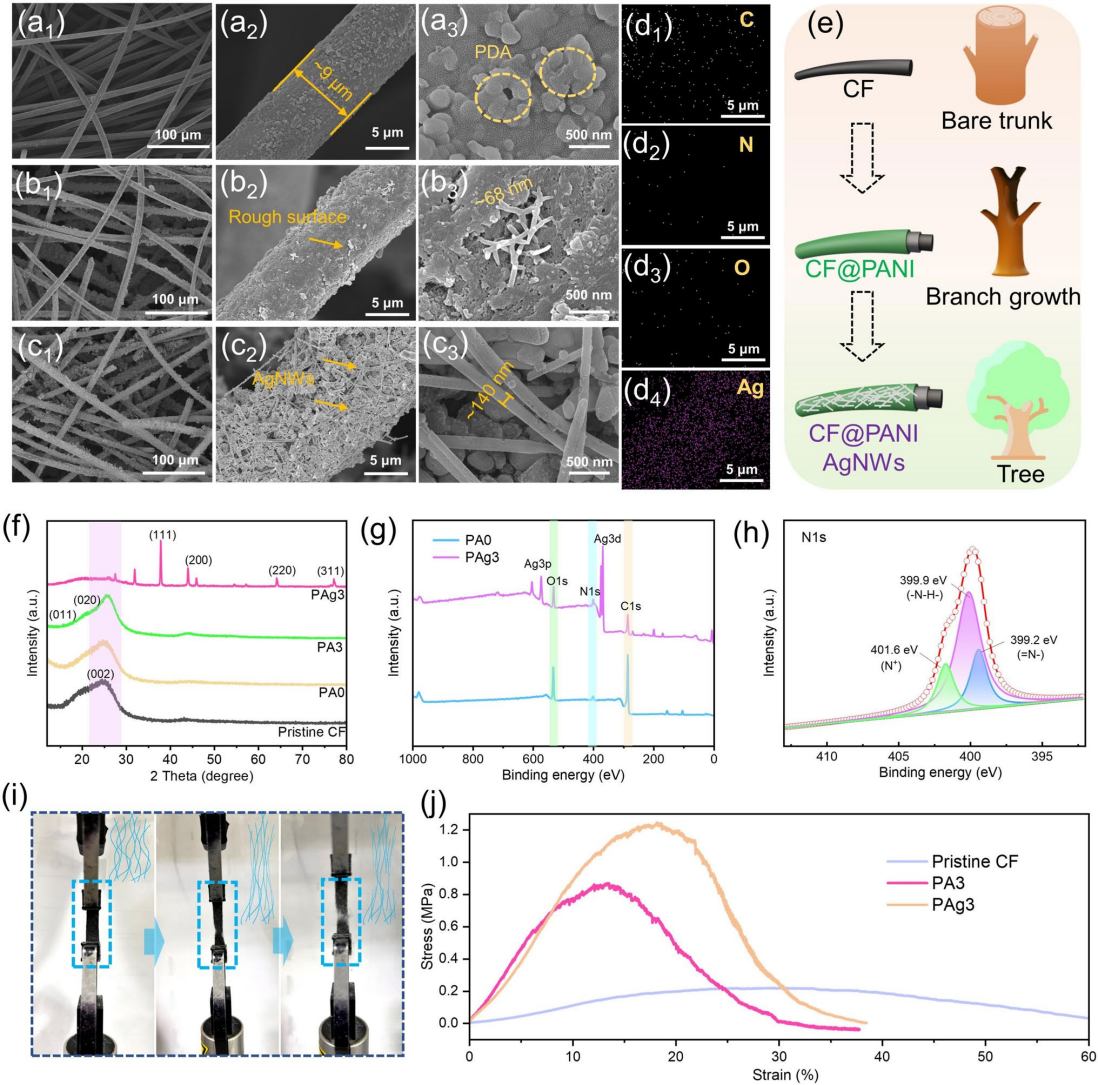
SEM images with different resolutions of **a** PA0, **b** PA3, **c** PAg3. **d** EDS mapping of PAg3, including the elements of C, N, O, and Ag. **e** Illustration of the branch-trunk interlocked structure of the PAg3. **f** XRD spectra of the prepared samples. **g** The total XPS spectra of PA0 and PAg3. **h** N 1*s* XPS spectrum of PAg3. **i** Mechanical tensile processes of the sample (the inserted blue lines represent the changes of carbon fibers during stretching). **j** Strain–stress curves of the different samples

Figure 2a, b, c present the changes in the surface micro-morphology of CF at different reaction processes. From Fig. S1a, b, one can find that the surface of pristine carbon fiber was smooth without impurities, and the diameter of carbon fiber was about 9 μm, as confirmed by the microscopic pictures (Fig. S1c, d). After modification with PDA, the surface of carbon fiber became rough (Fig. 2a_1–2_) and some agglomerated particles could be observed (Fig. 2a_3_) [34]. To improve the electric and mechanical properties of CF, PANI was selected for in-situ polymerization on the pretreated CF. Notably, the PDA with rich oxygen-containing groups would promote the attachment of PANI to CF through hydrogen bonding and π -π conjugation effect [35]. As shown in Fig. 2b_1–2_, the PANI layer made CF surface rougher. And the PANI nanowires with a diameter of ~ 68 nm could be seen from the high-resolution SEM image (Fig. 2b_3_). It is worth noting that the polymerization time plays an important role in the morphology of PANI. As can be seen in Fig. S4, it is observed that PANI fibers were wound on the surface of CF when the polymerization time was short (Fig. S4a, b). After the polymerization time was extended to 4 h, the PANI layer became compact and the fibrous structure disappeared (Fig. S4c). In addition to the influence on morphology, the polymerization time also had a great impact on the state of PANI. As we all know, PANI will change from one state to another in the oxidation process, which can be summarized as reduction state, doping state, and oxidation state [36]. Among them, the doped PANI can obtain outstanding electric conductivity, especially PANI doping in intermediate half oxidation states (emeraldine). In the process of doping, there is no electron gain or loss in the macromolecular chain of PANI, and the added protons bring the carriers needed for conduction to the macromolecular chain [37]. To further enhance the electric property and reduce the infrared emissivity of the composite, the AgNWs were sprayed on the PANI layer (Fig. 2c_1–3_). The results of energy dispersive spectroscopy (EDS) mapping also confirmed the existence of C, N, O and Ag (Fig. 2d_1–4_). Moreover, because the AgNWs were covered on the surface of composite, the element mapping showed a strong distribution of Ag.

**Fig. 3:**
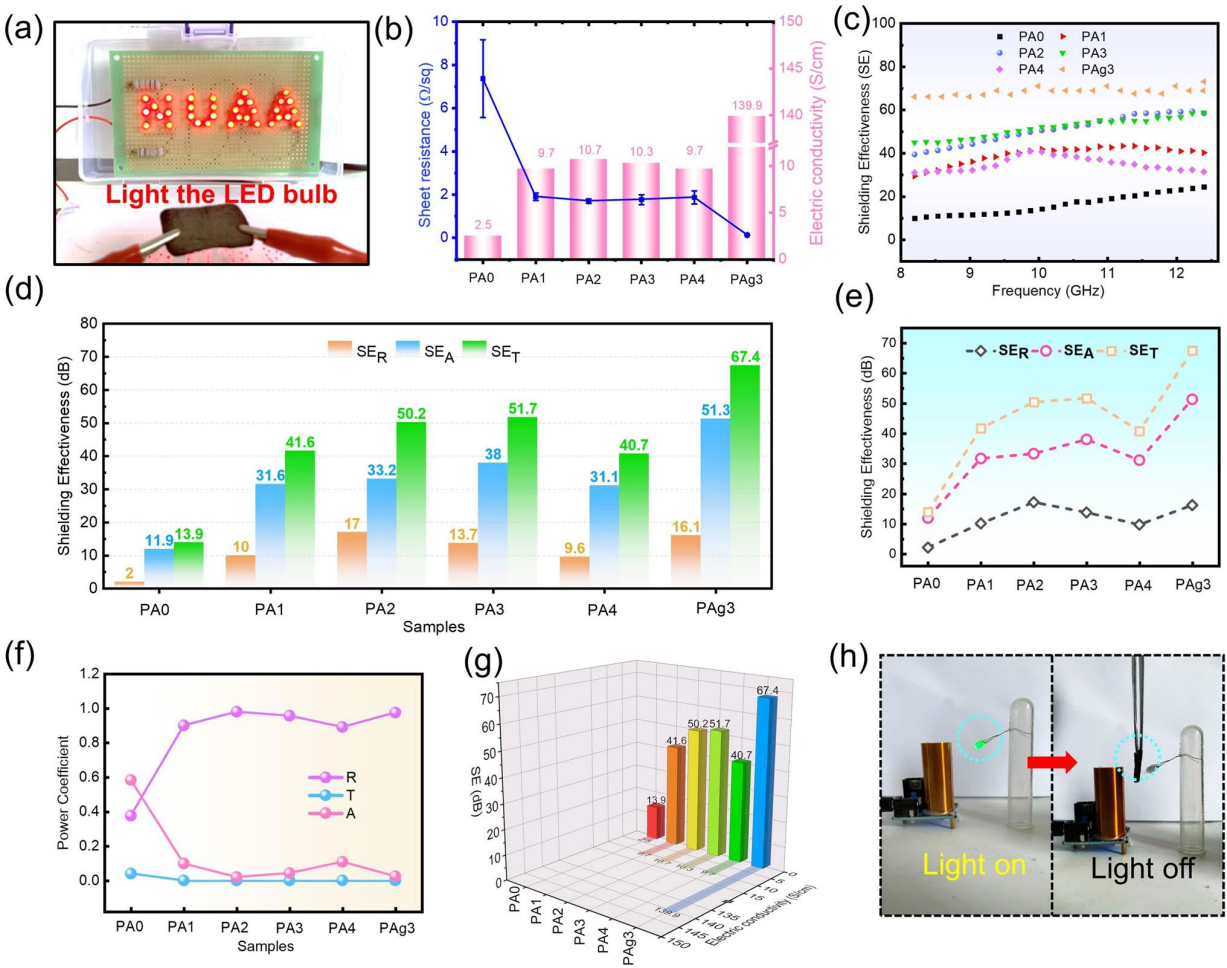
**a** Digital of PAg3 in closed circuits, and the LED bulb could be lighted up. **b** Square resistance and corresponding electric conductivity of the samples. **c** EMI shielding properties of the different samples. **d, e** Proportion of SE_A_, SE_R_, and SE_T_ of the samples at 10 GHz. **f** Power coefficients of the samples, including A, R, and T. **g** Relationship between EMI shielding property and electric conductivity of the samples at 10 GHz. **h** Photograph of Tesla coil used to show EMI shielding performance of PAg3

**Fig. 4:**
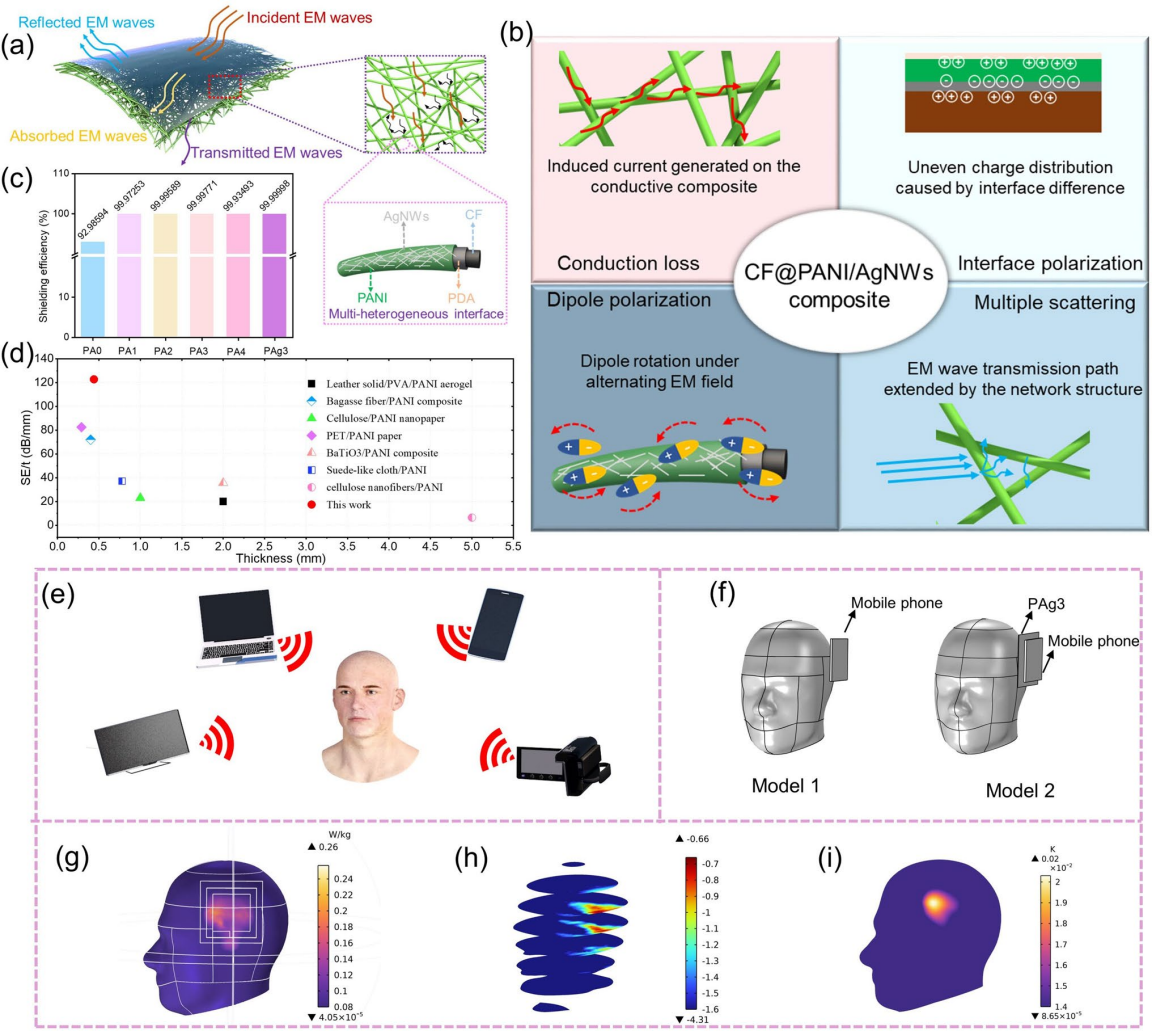
**a** Schematic diagram of when the EM waves were irradiated on the sample. **b** Possible EM waves attenuation mechanism of the sample. The conduction loss, interface polarization, dipole polarization, and multiple scattering contribute together to EM wave absorption. **c** Shielding efficiency of the prepared samples. **d** Comparisons of EMI shielding performances in this work with the reported works. **e** Illustration of the influence of electronic equipment on human beings. **f** Established models for simulations. Model 1: a mobile phone is placed next to the human brain; Model 2: based on Model 1, the PAg3 was inserted between the phone and the brain. **g** SAR of the brain under the irradiation of EM waves (Model 2). **h** Sectional view of SAR of the brain (Model 2). **i** Temperature change of the brain after being irradiated by EM waves (Model 2)

The XRD spectra were applied to investigate the composition of the prepared composites. As shown in Fig. 2f, the pristine CF displayed a broad peak at about 26°, corresponding to the (002) plane of amorphous carbon. The Raman spectrum of CF was exhibited in Fig. S5. The two peaks located in ~ 1350 (*I*_D_) and ~ 1580 (*I*_G_) cm^−1^ could be seen, which was attributed to the disordered carbon and graphitized carbon, respectively [38, 39]. The high value of *I*_D_/*I*_G_ (0.973) indicated the high defect degree of carbon. In addition, the peaks at 18.2° and 26.6° represent the intrinsic PANI and the periodic arrangement of PANI molecular chains in the vertical direction, respectively, demonstrating good structural regularity and crystallinity [40]. And the XRD spectrum of PAg3 also showed typical diffraction peaks of AgNWs. The above analysis proved that the composite was successfully prepared. The XPS spectra were used to further discuss the element state of the composites (Fig. 2g). From the wide scan spectrum, C (285.1 eV), Ag (368.1 eV), N (400.1 eV), and O (532.1 eV) could be found in PAg3, in agreement with the EDS results. In Fig. S6a, the C 1*s* can be decomposed into two types of carbon species: C–C and C–O, corresponding to 284.8 and 287.9 eV. O 1*s* scan spectrum (Fig. S6b) exhibits two distinct peaks at 531.5 eV (C–O) and 532.4 eV (C=O). Two main peaks appear at 368.1 and 374.2 eV in the XPS spectrum of Ag 3*d* (Fig. S6c). Moreover, N 1*s* (Fig. 2h) is fitted into 399.2 eV (= N–), 399.9 eV (–N–H–), and 401.6 eV (N^+^), showing that protons were doped on half oxidation PANI [41, 42]. Interestingly, the synthesis process of composite is like a tree growing (Fig. 2e). The CF was used as the trunk for PANI (branch) growth. And the PDA could bond them to form a tight and stable interlocking structure. The addition of AgNWs made the whole “tree” more complete and prosperous, improving the function of the prepared composite. Benefiting from this branch-trunk interlocked structure, the mechanical property of the composite was greatly enhanced. And the intertwining fiber network of the composite further boosted its tensile properties (Fig. 2i). In the process of stretching, the loose carbon fibers were elongated at first, and then gradually broke during continuous stretching (as shown by the blue lines inserted in Fig. 2i), which ensured that PAg3 will not break directly. Consequently, the tensile strength increased from initial ~ 0.2 MPa for pristine CF to ~ 0.84 MPa for PA3, which was almost 4 times as much as pristine CF. Additionally, the tensile strength of PAg3 could reach about 1.2 MPa (Fig. 2j), showing superior mechanical performance.

### EMI Shielding Performance of the Composites

At present, highly efficient EMI shielding materials are required to regulate the unwanted EM waves, so as to reduce the adverse impact on human society. Without considering the structure of the material, electric conductivity plays a dominant role in the EMI shielding performance of the material [43]. As displayed in Fig. 3a, when the sample was connected to the circuit, it can light the bulb with “NUAA”. And the other samples can also light the bulb (Fig. S7a-e), indicating that all samples had certain conductivity. The four-point probe method was employed to further analyze the square resistance and conductivity of samples, which can be seen in Fig. 3b. The conductivity of pristine CF was about 2.5 S cm^−1^. After the polymerization of PANI, the conductivity was greatly enhanced to 9.7 S cm^−1^ when the polymerization time was 1 h. And then the conductivity reached its maximum within 2 or 3 h of polymerization time. Nevertheless, If the polymerization time continued to be prolonged, the conductivity of the sample showed a downward trend. This phenomenon was mainly related to the oxidation state of PANI, which verified the previous analysis, that is, PANI doped with intermediate half oxidation states could obtain good conductivity. What’s more, the sprayed AgNWs further improved the conductivity of the sample to 139.9 S cm^−1^. The high conductivity ensured the excellent EMI shielding performance of the prepared samples. From Fig. 3c, it can be found that the PA0 possessed an average EMI shielding performance of 16.2 dB. While the EMI shielding performance of PA2 and PA3 could reach about 51.7 dB. Notably, the maximum shielding property of 73.9 dB was achieved for PAg3. The SE_R_, SE_A_, and SE_T_ of all samples at 10 GHz are revealed in Fig. 3d, e. It is observed that the SE_A_ always occupied the SE_T_, which proved that most of the EM waves incident inside the material were attenuated by absorption. And this reduced the secondary reflection pollution of EM waves to some extent. However, it is insufficient to judge the EMI shielding mechanism of samples by only comparing the values of SE_R_ and SE_A_. Therefore, the power coefficients of the samples, including A, R, and T are demonstrated in Fig. 3f. Except for PA0, other samples had relatively high R values, suggesting that the dominant EMI shielding mechanism of samples was reflections. This was mainly due to the high conductivity of the samples, which led to the EM waves being reflected before it was incident inside the samples [44]. Figure 3g shows the relationship between shielding performance and conductivity of the sample at 10 GHz. And it once again proves the important influence of conductivity on shielding performance. In order to observe the shielding performance of the PAg3 more directly, the tesla coil was applied as shown in Fig. 3h and Video S1. The LED bulb could be lit up due to the EM induction phenomenon. While the LED light would go out when the PAg3 was inserted between the bulb and the coil [45].

According to the transmission line theory, the EM wave transmission process in the sample is demonstrated as follows (Fig. 4a). First of all, most EM waves will be reflected when they incident on the surface of the sample. And this is mainly due to the impedance imbalance between the samples and free space caused by high electric conductivity [46, 47]. Second, residual EM waves will enter the interior of the samples. In this stage, the EM waves will be attenuated and converted into thermal energy through conduction loss, dipole polarization, and interface polarization from multiple heterogeneous interfaces (Fig. 4b) [48–51]. More importantly, the conductive fiber network structure of the sample will lead to the multiple scattering behaviors of EM waves, which greatly prolongs the transmission path of EM waves, thus further enhancing the microwave absorption property of the sample [52]. The shielding efficiency of samples was evaluated by the following equation:

7 $$Shielding efficiency \left ( \% \right) = 100 - \left( {1/10^{SE/10} } \right) \times 100$$

When the SE is greater than 20 dB, it means that 99% of EM waves are effectively shielded. As exhibited in Fig. 4c, the PAg3 can block more than 99.999% of EM waves, and it once again proves its powerful EMI shielding ability. To highlight the superiority of this work, Fig. 4d compares the properties of PANI-based shielding materials with different thicknesses [53–59]. It can be found that the PAg3 shows outstanding EMI shielding performance at low thicknesses. As we know, the rapid development of electronic devices, such as mobile phones, laptops, smart TV, and so on, makes the EM environment around human beings increasingly complex, thereby having a negative impact on human health (Fig. 4e). And the main hazards of EM waves come from the “thermal effect” produced by polar molecules in the human body, which can be evaluated by the specific absorption rate (SAR). SAR refers to the electromagnetic radiation energy absorbed by a unit mass of matter per unit time. To this end, a multi-physical field simulation via Comsol software was carried out to observe the EMI shielding effect of the synthesized samples on the electronic equipment we use every day. As shown in Fig. 4f, two models were built (Model 1 and Model 2), in which one model was not inserted with PAg3 and the other model was, respectively. From Figs. 4g, h and S9a, b, it could be found that the maximum SAR of Model 1 was about 2.29 W kg^−1^. While the SAR of Model 2 was only 0.26 W kg^−1^. At the same time, the radiation depth of Model 2 to the brain was also relatively small. In addition, the EM waves will produce thermal effects on human tissues. Figures 4i and S9c indicate the temperature changes of different models. When Model 1 was exposed to EM radiation, its temperature increased by 0.15 K. In comparison, the temperature value of model 2 was increased by 0.02 K, showing great EM wave-blocking ability.

### Thermal Management of the Composites

**Fig. 5:**
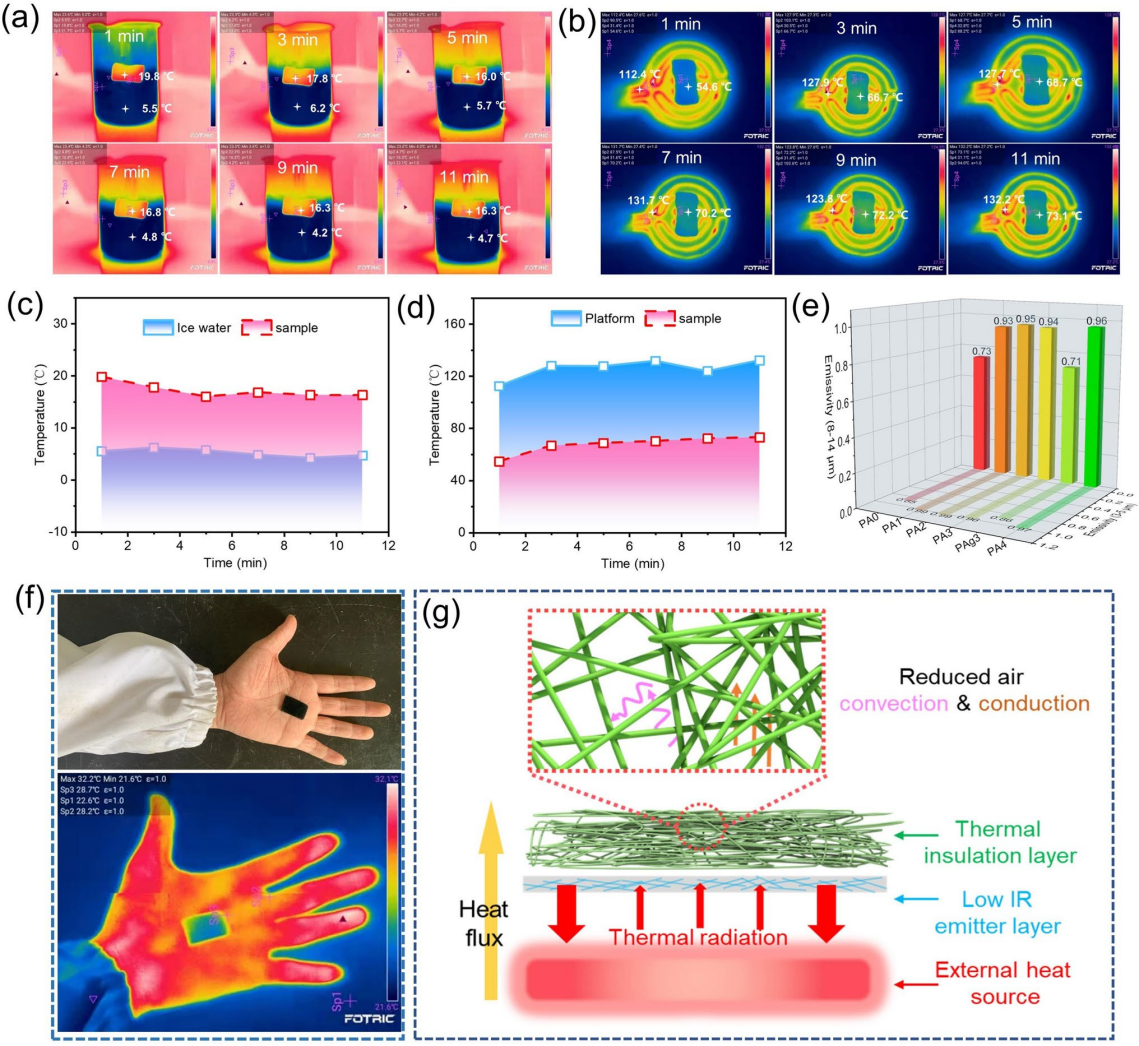
**a** Infrared images at different times when the PAg3 was attached to the surface of the ice water bath. **b** Infrared images at different times when the PAg3 was placed on the heated platform. **c** Temperature change of PAg3 and ice water bath. **d** Temperature change of PAg3 and heat platform. **e** Infrared emissivity of the prepared composites at 3–5 μm and 8–14 μm. **f** Infrared stealth ability of the PAg3, the sample was placed on the hand to observe the infrared difference between the surface of the sample and the hand. **g** Mechanisms of the sample thermal insulation and infrared stealth

Benefiting from a unique multi-level design, the PAg3 possesses superior thermal insulation performance, showing potential as a wearable device in cold areas. As seen in Fig. S10a, the PAg3 was attached to a cup containing a mixture of ice and water, and the temperature change and infrared images were recorded in this process. From Fig. 5a, it is found that its surface temperature could be maintained at about 16 ℃ even if it was left for a long time, which was close to room temperature (~ 20 ℃). The temperature change tendency can be observed more clearly in Fig. 5c. Apart from heat preservation in the civil field, the PAg3 could also be used in the field of infrared stealth. The PAg3 was placed on the heated platform (~ 120 ℃) and the temperature changes of the heating platform and sample surface were monitored (Fig. S10b). The surface temperature of PAg3 was about 54.6 ℃ at 1 min. With the extension of heating time, the surface temperature of PAg3 was gradually increased and stabilized at about 73 ℃ (Fig. 5b). Figure 5d indicates that the temperature-rising trend of the sample surface was very slow, demonstrating an exceptional heat insulation effect of PAg3. According to Stefan-Boltzmann's law [60, 61], controlling temperature and infrared emissivity can effectively reduce the radiation energy density of objects, thus achieving the goal of infrared stealth. Therefore, the infrared emissivity of all samples at 3–5 μm and 8–14 μm was comprehensively investigated as shown in Fig. 5e. It is worth noting that the PAg3 had quite low infrared emissivity, wherein the emissivity was 0.86 in the range of 3–5 μm and 0.71 in the range of 8–14 μm, which can be attributed to the high conductive AgNWs layer. When the PAg3 was placed on the hand (the side of the sprayed AgNWs faced upwards), it can be clearly observed that the PAg3 was able to cover the infrared radiation of the hand (Fig. 5f), demonstrating its great infrared stealth feature. The brilliant heat preservation and infrared stealth performances of the PAg3 mainly come from special components and structural design, which is illustrated in Fig. 5g. On the one hand, the fluffy multi-fibrous structure endows the prepared composite with superior thermal insulation properties. From Fig. S11, we can find that the thermal conductivity of PAg3 was only 0.1031. And the thermal conductivity of pristine CF and PA3 was 0.1018 and 0.1044, respectively, indicating that the thermal conductivity of the PAg3 was basically not affected by the modification. Thus, this structure can reduce air conduction and convection as much as possible, and prevent heat from escaping. On the other hand, the AgNWs layer on the surface of PAg3 can efficiently block the infrared radiation, making the sample have low infrared emissivity. All these characteristics of heat management make the PAg3 very suitable for various scenarios.

**Fig. 6:**
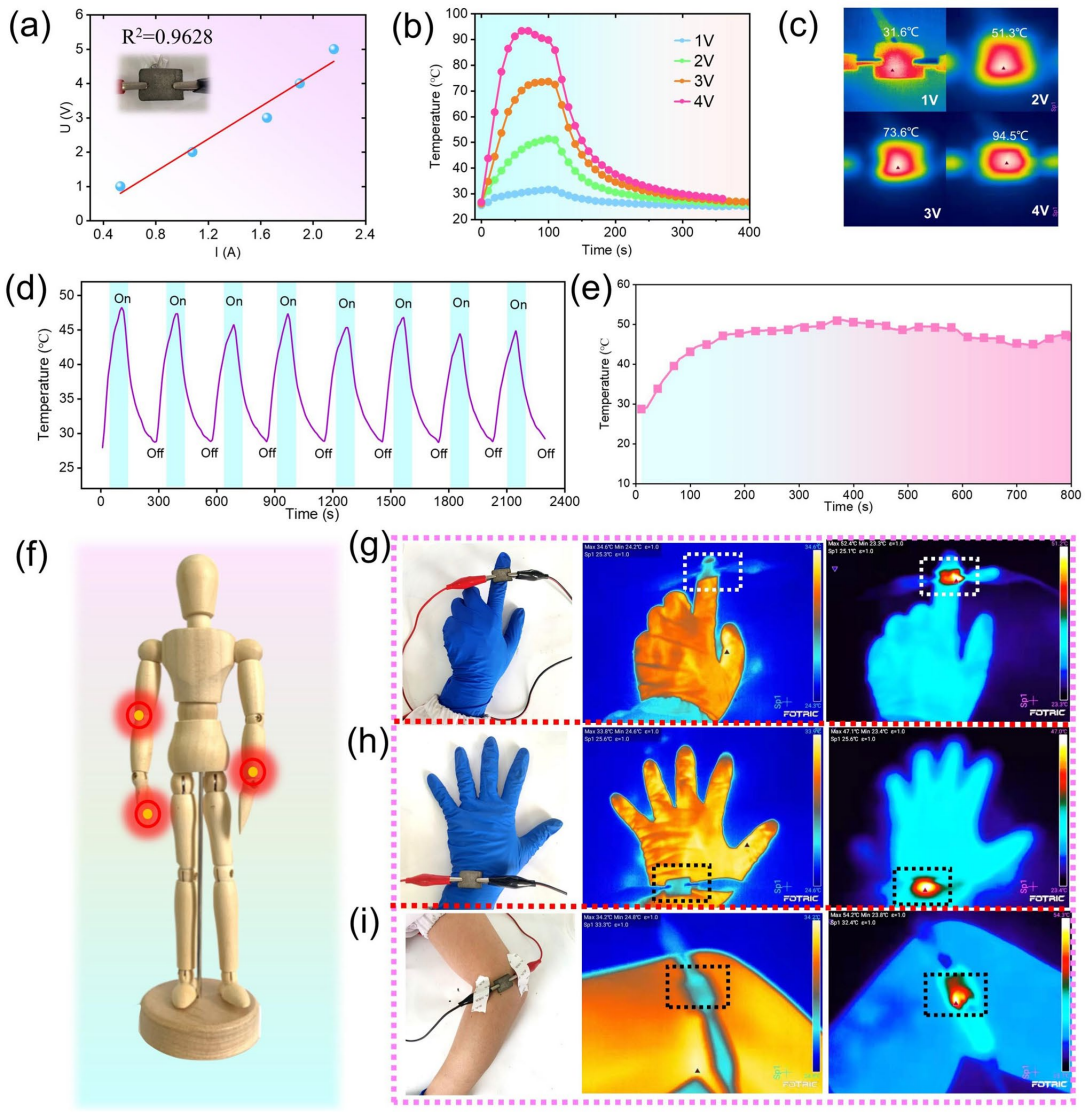
**a** *V-I* linear curve of the PAg3. **b** Surface temperature changes and **c** corresponding infrared images of PAg3 with varied input voltages. **d** Electric-to-heating cycle stability of PAg3 at 2 V. **e** Durability of sample electrothermal at 2 V. **f** Diagram of electrothermal properties of PAg3 for human therapy. The infrared images of the PAg3 at 2 V when it was attached on the **g** figure, **h** wrist, and **i** elbow

In addition to the passive thermal management, the PAg3 can also actively control the temperature through electro-thermal conversion (*i.e*., Joule thermal effect) due to its good conductivity. Herein, the Joule heating performance of PAg3 was investigated in detail and the size of the PAg3 to which the voltage was applied was 2.5 cm × 1.5 cm. Figure 6a displays the linear *I-V* curve of the PAg3, which completely follows Ohm's law (R^2^ = 0.9628). Additionally, the U^2^-temperature curve is also highly in line with Joule’s Law (Q = U^2^t/R, R^2^ = 0.9626) as can be seen in Fig. S12, suggesting that it can be used as an electric heater with a high heating operability by adjusting the input direct voltage [62]. The specific Joule heating property of PAg3 was estimated with a fixed direct voltage from 1 to 4 V, and the temperature change of the sample surface was recorded with a thermocouple probe and infrared images were taken at the same time with the FOTRIC infrared camera (Fig. 6b, c). It is obviously observed that the surface temperature of the sample increased with the increase of input voltage, and it gradually stabilized at the saturation temperature, showing the facile and accurate operation of Joule heating performance. When the applied voltage was 4 V, the saturation temperature can reach 93.3 ℃ (Fig. 6b), exhibiting perfect low-voltage Joule heating performance. Moreover, stability and durability are also important criteria for evaluating Joule heating properties of materials, which can not be ignored. Figure 6d shows the cyclic heating/cooling process of PAg3 at a fixed voltage of 2 V, proving its great long-range stability. Meanwhile, the temperature of the PAg3 can remain stable under the condition of heating for a long time with an applied voltage of 2 V (Fig. 6e), which also indicates the durability of Joule heating. The good Joule heating property of the PAg3 makes it available as a wearable device, which serves as a heat supply in cold regions and thermal therapy for certain diseases (for example, rheumatism) (Fig. 6f–i).

### Super-hydrophobic and Self-Cleaning Properties of the Composite

**Fig. 7:**
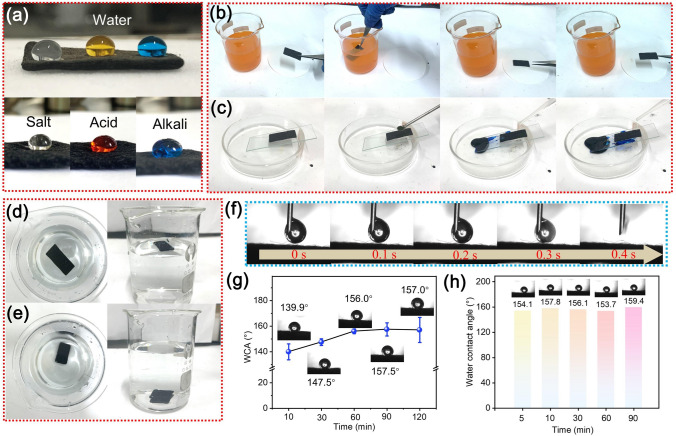
**a** Digital photos of the superhydrophobic properties of the sample. **b, c** Self-cleaning performance of the superhydrophobic composite. The state of the sample in water **d** with and **e** without hydrophobic treatment. **f** Water contact angle of the sample without hydrophobic treatment. **g** Changes in the sample contact angle with the time of hydrophobic treatment. **h** Stability of superhydrophobic property of the sample

In order to improve the practicability of the prepared composite and ensure its stable performance during use. A dip-coating method was used to form a superhydrophobic PDMS layer on the surface of the composite. As shown in Fig. 7a, water droplets can exist stably on the surface of the composite without infiltrating it. And it is noted that the acid, alkali, and salt solution can remain stable when dripping on the surface of the composite, indicating great corrosion resistance. In comparison, water droplets quickly entered the composite without hydrophobic treatment (Fig. 7f). Figure 7d, e show that the hydrophobic composite could easily float on the water, while the composite without a PDMS layer would sink directly to the bottom of the water due to the existence of a large number of active groups. And the hydrophobic feature will endow the composite with excellent self-cleaning performance. Figure 7b displays that when the hydrophobic composite was immersed in methyl orange solution, it basically didn't stick to any dye solution. In addition, impurities on its surface can be easily removed by washing it with water (Fig. 7c). The effect of PDMS treatment time on the hydrophobic properties of the composite was investigated in Fig. 7g. One can find that with the prolongation of treatment time, the hydrophobic properties of the composites became better and better, and finally reached stability. When the treatment time was 60 min, the composite reached a superhydrophobic state with a WCA of ~ 156.0°. What’s more, this superhydrophobic property of the composite can be maintained for a long time (Fig. 7h), demonstrating its outstanding stability. The excellent water resistance of the composite is mainly attributed to the following points: (1) the PDMS layer makes the surface of the material have low surface energy; (2) the unique micro/nanostructure endows the composite rough surface, decreasing the water–solid contact area and thus enhancing hydrophobicity [63, 64].

## Conclusions

In summary, the CF@PANI/AgNWs composite was successfully synthesized through the assembly of multiple 1D materials. And the strong adhesion of PDA ensures the stability of the unique branch-trunk interlocked micro/nanostructure, endowing the composite enhanced mechanical property. Meanwhile, the synergistic effect of low thermal conductivity and emissivity made the composite possess exceptional thermal control ability. This feature, of course, can also be used in infrared stealth. More importantly, due to the conductive network constructed by multiple 1D materials, the obtained composite demonstrated the superior EMI SE of 73.9 dB and Joule heating performance with a saturation temperature of 93.3 ℃ at 4 V low applied voltage. It is noted that the superhydrophobic trait of the composite is conducive to being used in more complex environments. All the great properties the composite present make it have great potential for a dual-function wearable device with efficient thermal energy management and electromagnetic protection.

### Supplementary Information

Below is the link to the electronic supplementary material.

Supplementary file1 (MP4 1190 kb)

Supplementary file2 (PDF 992 kb)
