# Supplementary material for: "Three-in-One" Multi-Scale Structural Design of Carbon Fiber-Based Composites for Personal Electromagnetic Protection and Thermal Management
Source: Nanomicro Lett. 2023 Jul 10;15:176. doi: 10.1007/s40820-023-01144-z (PMC10333170; doi:10.1007/s40820-023-01144-z)
Supplement: Supplementary file 2 — Supplementary file2 (PDF 992 kb) [file 40820_2023_1144_MOESM2_ESM.pdf]

Supporting Information for

"Three-in-One" Multi-scale Structural Design of Carbon Fiber-based Composites for Personal Electromagnetic Protection and Thermal Management

Ming Zhou[1], Shujuan Tan [1,*], Jingwen Wang[1], Yue Wu[1], Leilei Liang[2], Guangbin Ji[1]

[1] College of Materials Science and Technology, Nanjing University of Aeronautics and Astronautics, No. 29 Yudao Street, Nanjing 210016, P.R. China

[2] School of Electronic Science and Engineering, Nanjing University, Nanjing, 210093 P. R. China

*Corresponding author. E-mail: tanshujuan@nuaa.edu.cn (Shujuan Tan)

Supplementary Figures and Tables

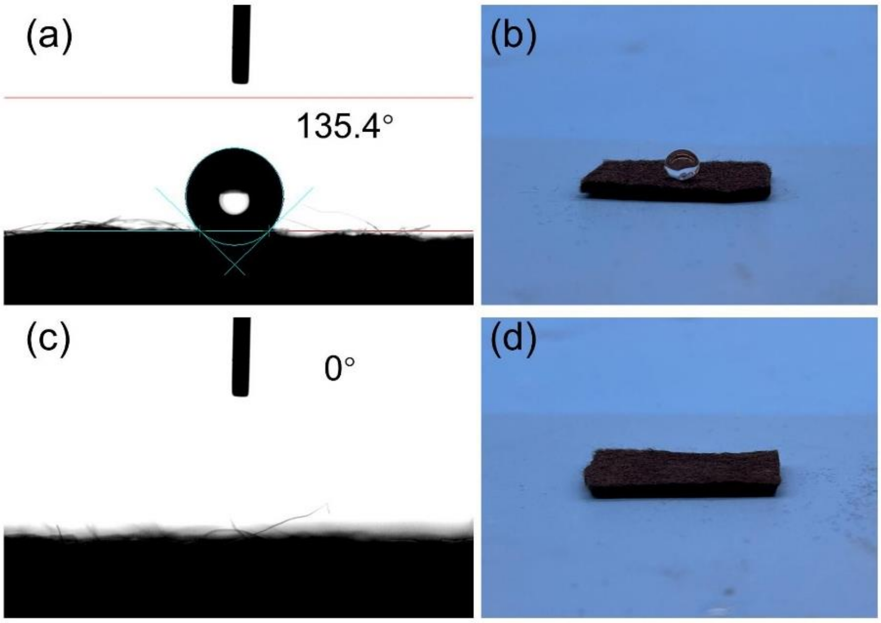

Fig. S1 WCA of the pristine CF (**a**) and the PDA modified CF (**c**). The digital images of water on the pristine CF (**b**) and the PDA modified CF (**d**)

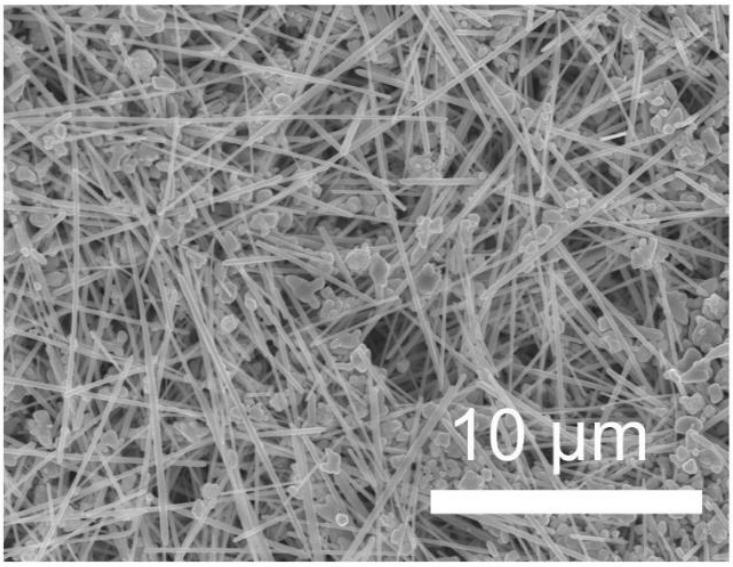

Fig. S2 SEM image of the synthesized AgNWs

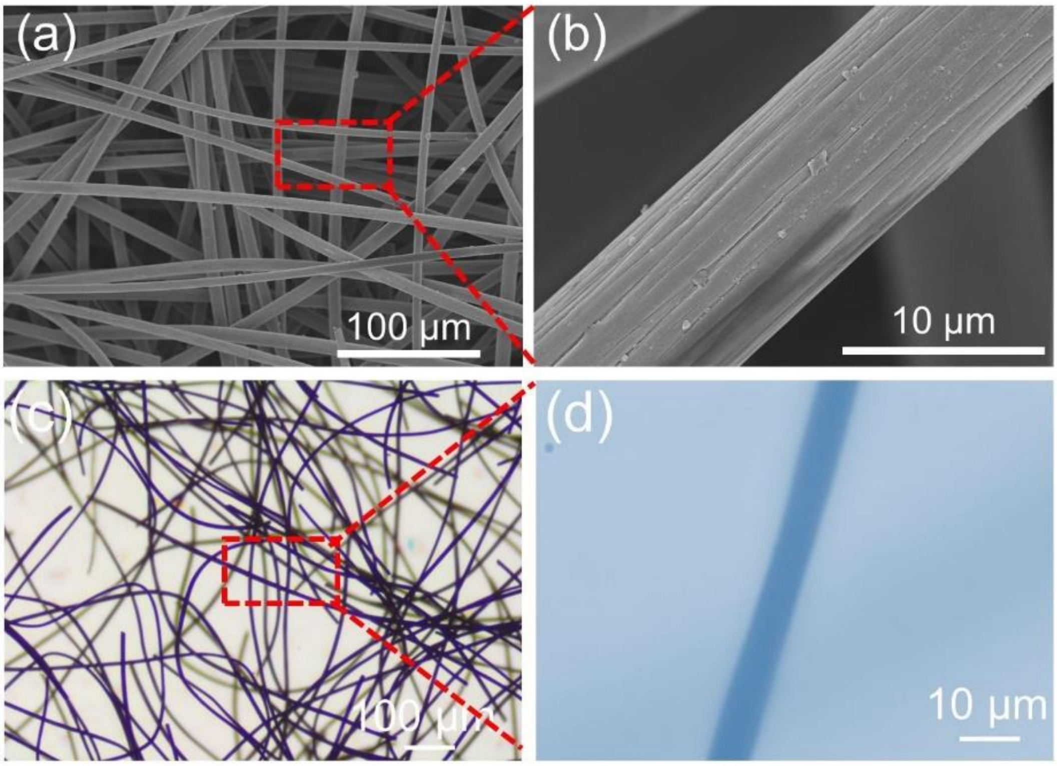

Fig. S3 SEM images of the pristine carbon fiber with different magnifications (**a, b**). Microscopic photographs of the pristine carbon fiber with different magnifications (**c, d**)

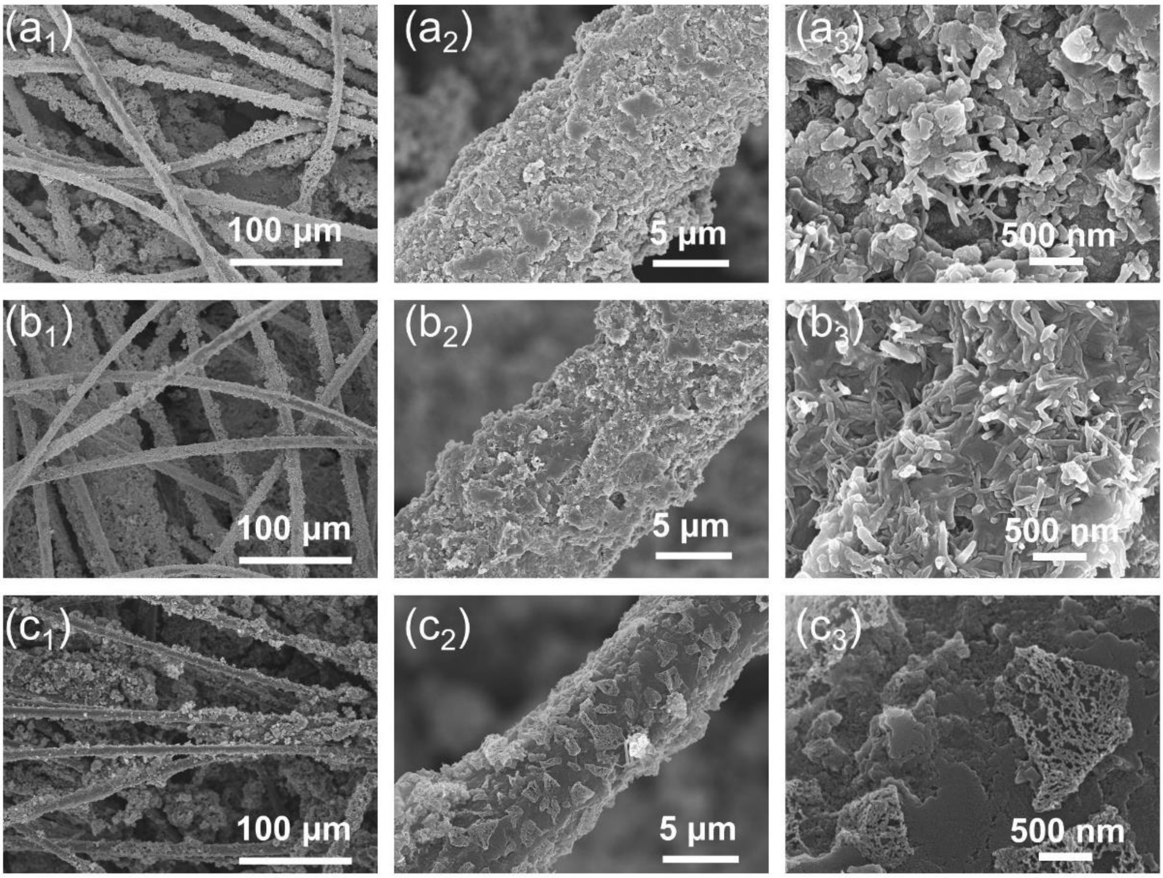

Fig. S4 SEM images with different resolutions of (**a**) PA1, (**b**) PA2, and (**c**) PA4

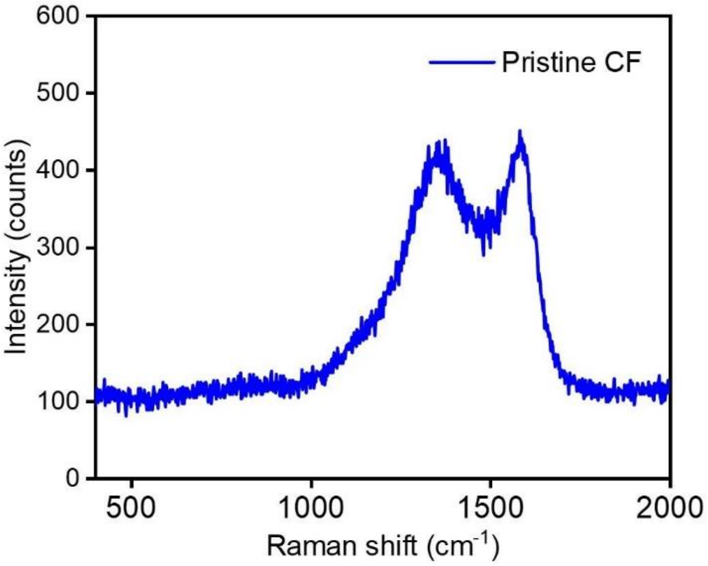

Fig. S5 Raman spectrum of pristine CF

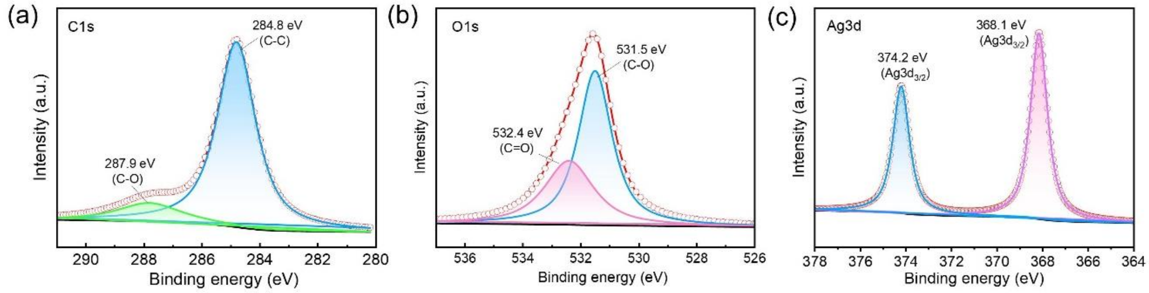

Fig. S6 C 1s (**a**), O 1s (**b**) and Ag 3d (**c**) XPS spectrum of PAg3

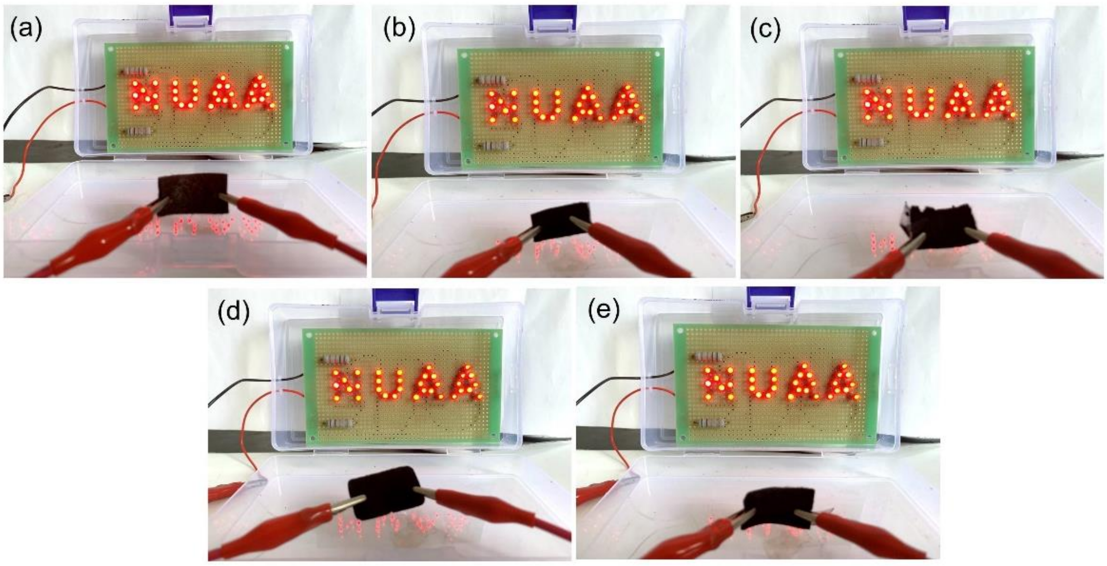

Fig. S7 Digital of PA0 (**a**), PA1 (**b**), PA2 (**c**), PA3 (**d**) and PA4 (**e**) in closed circuits

Simulation of Specific absorption rate (SAR): Comsol Multiphysics software (version of 6.0) was employed to observe the influences of electromagnetic radiation on the human brain. And the SAR is usually evaluated by the following formula:

$$E_{SAR} = \sigma \frac{|E|^2}{\rho}$$

Where σ is denoted as the electric conductivity of the human tissue and ρ is the density. E is the effective value of spatial field strength in human tissue, it can be expressed by the formula:

$$E = \sqrt{E_x^2 + E_y^2 + E_z^2}$$

To simulate the impact of electronic devices used in daily life, for example, the mobile phone on the human brain, a model, including the human brain and a microstrip patch antennae, was built, where the antenna was 0.1m to the left of human brain. It is noted that the radiation frequency was set as 0.853 GHz, which is the frequency band

commonly used for cell phone communication. The heat change of human brain under antenna radiation was analyzed in the process of simulation. The excitation mode of electromagnetic field was selected as the Lumped Port. In addition, to eliminate unnecessary scattered radiation, Perfectly Matched Layer (PML) was chosen as the boundary of the model (Fig. S8). And this model solves the vector-Helmholtz equation for a given frequency everywhere in the domain:

$$\nabla \times \frac{1}{\mu_r} \nabla \times E - k_0^2 \varepsilon_r E = 0$$

Where μ_r and ε_r represent relative permeability and raltive permittivity, respectively. And k_0 is the wave vector of free-space.

The parameter setting of the human brain was based on the institution standards of IEEE and IEC (For more specific parameters, please refer to Comsol's official website). The parameters used in this model are shown in the following table:

Table 1 The parameters of the model

Component	Value	unit
Permittivity of brain tissue	58.13	-
Conductivity of brain tissue	1.15	S/m
Density of brain tissue	1030	kg/m^3
Specific heat capacity of blood	3639	J/(kg·K)
Density of blood	1000	kg/m^3
Permittivity of patch antenna	5.23	-

The physical fields of biological heat transfer and Electromagnetic wave were chosn to calculate the change of temperature with electromagnetic radiation.

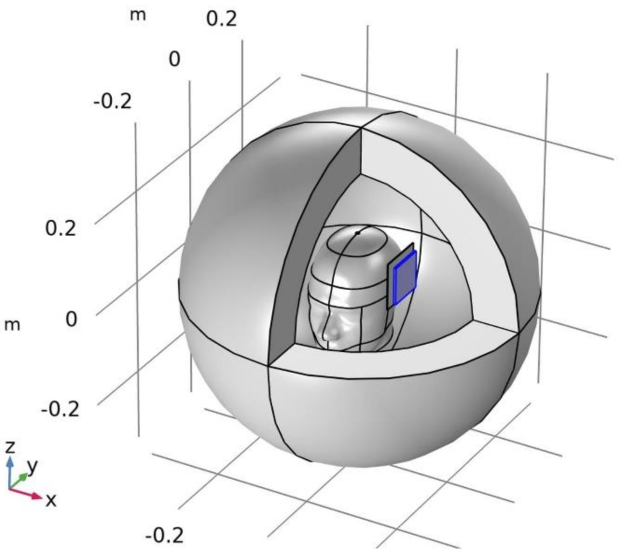

Fig. S8 The model of about PAg3 surrouding by PML

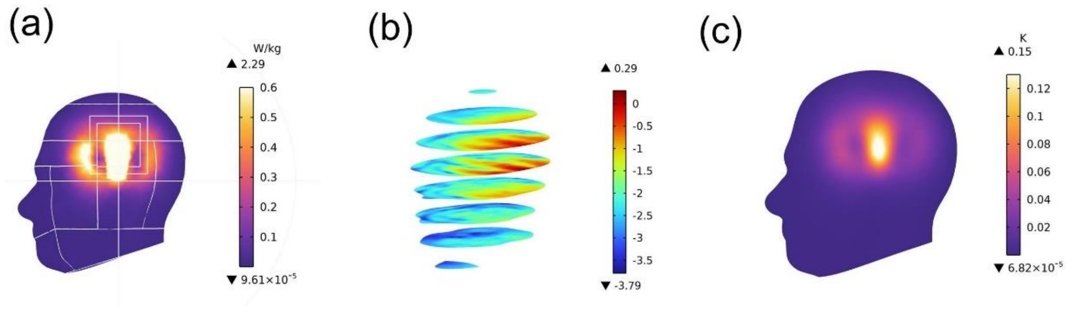

Fig. S9 (**a**) SAR of the brain under the irradiation of EM waves (Model 1). (**b**) Sectional view of SAR of the brain (Model 1). (**c**) The temperature change of the brain after being irradiated by EM waves (Model 1)

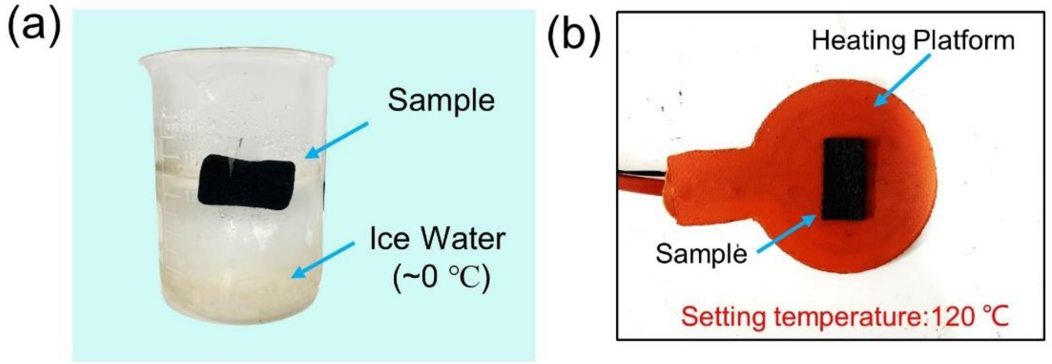

Fig. S10 Digital iamges of the PAg3 attached on the ice water (**a**) and placed on the heating platform (**b**)

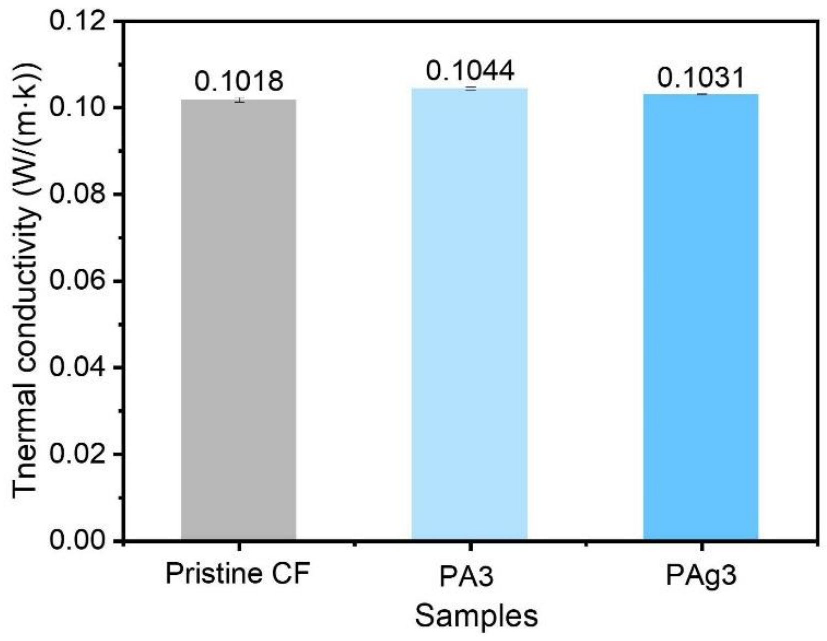

Fig. S11 The thermal conductivity of the samples

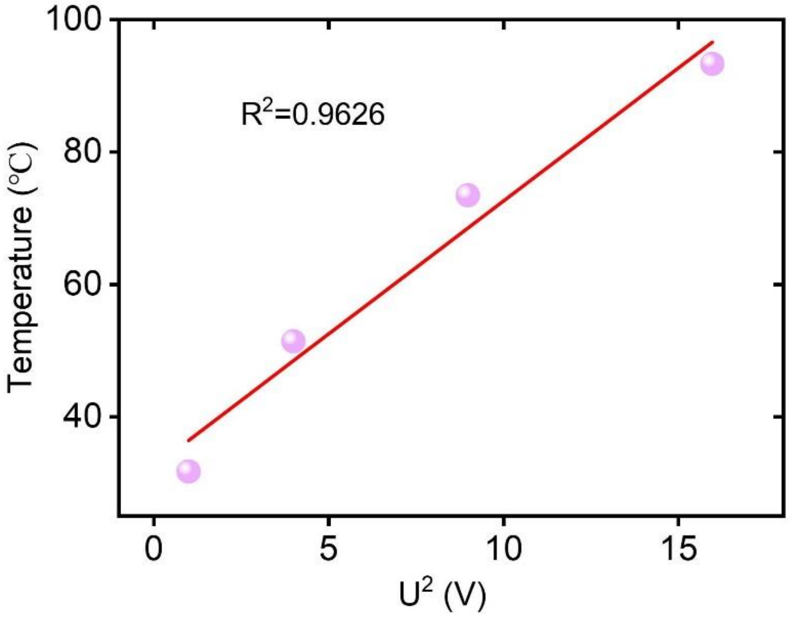

Fig. S12 T-U^2 curve of the PAg3
